# hUC-MSC extracellular vesicles protect against hypoxic-ischemic brain injury by promoting NLRP3 ubiquitination

**DOI:** 10.17305/bb.2024.10706

**Published:** 2024-12-26

**Authors:** Shanshan Xiao, Ying Lv, Xuejing Hou, Shuqiang Qu

**Affiliations:** 1 Department of Pediatrics, The Second Affiliated Hospital of Harbin Medical University, Harbin, China; 2 Department of Pediatrics, The Fourth Affiliated Hospital of Harbin Medical University, Harbin, China

**Keywords:** Mesenchymal stem cells, MSCs, extracellular vesicles, EVs, hypoxia-ischemia, HI, brain, brain injury, neuroprotection, NOD-like receptor family pyrin domain-containing 3, NLRP3, inflammasome, microglia, ubiquitination, pyroptosis, protein kinase A, PKA

## Abstract

Hypoxic-ischemic brain injury (HIBD) is a major cause of neonatal mortality and long-term neurological deficits, with limited treatment options. Extracellular vesicles (EVs) from human umbilical cord mesenchymal stem cells (hUC-MSC-EVs) have shown promise in neuroprotection, but the mechanisms remain unclear. This study explores how hUC-MSC-EVs protect neonatal rats from HIBD. hUC-MSC-EVs were isolated, characterized, and administered to neonatal rats subjected to HIBD. Behavioral reflexes and brain infarction were assessed, along with cellular and molecular analyses of hippocampal tissue. An *in vitro* oxygen–glucose deprivation/reoxygenation (OGD/R) model was used to simulate ischemic conditions in rat primary microglia. Results demonstrated that hUC-MSC-EVs significantly improved neurological outcomes, reduced brain infarction, and decreased microglial activation and pyroptosis. These effects were linked to the inhibition of NLRP3 inflammasome activation and enhanced ubiquitination via the protein kinase A (PKA) pathway. Blocking PKA partially reversed these protective effects. Here we highlight that hUC-MSC-EVs provide neuroprotection by regulating the NLRP3 inflammasome, offering a potential therapeutic strategy for HIBD. These findings expand the understanding of EV-mediated neuroprotection and suggest broader applications for ischemia-related conditions, with potential for clinical translation.

## Introduction

Hypoxic-ischemic brain injury (HIBD) is one of the most severe neurological conditions affecting neonates, contributing significantly to perinatal morbidity and mortality [1]. HIBD arises when the brain is deprived of adequate oxygen (hypoxia) and blood flow (ischemia), typically occurring during or around the time of birth [2]. Its prevalence varies globally, ranging from 1% to 8% in developed countries to as high as 26% in resource-limited settings [3, 4]. HIBD is a leading cause of lifelong disabilities, including motor dysfunction (e.g., cerebral palsy), sensory deficits, cognitive impairments, epilepsy, and other neurodevelopmental disorders [5]. These outcomes have profound implications, not only for affected individuals but also for their families and healthcare systems. Given the substantial global burden of HIBD, research into effective treatments remains a priority [6]. Currently, therapeutic options for HIBD are limited. Therapeutic hypothermia, which involves cooling the body to reduce metabolic demands and slow the cascade of brain injury, is the most widely used intervention [7]. Although hypothermia has demonstrated some efficacy in improving outcomes, its effects are only partial, and its application is often restricted to high-resource settings [8]. Moreover, many infants undergoing hypothermia therapy still experience significant neurological sequelae [7]. These limitations underscore the urgent need for novel, more effective neuroprotective therapies that are broadly accessible to mitigate the impact of HIBD. The mechanisms underlying HIBD are multifactorial, involving complex and interrelated pathological processes. Hypoxic-ischemic events trigger a cascade of damaging responses, including excitotoxicity, oxidative stress, mitochondrial dysfunction, and critically, neuroinflammation [3, 9]. These processes cause extensive damage to neurons and glial cells, particularly in regions like the hippocampus and cerebral cortex, which are highly vulnerable to ischemic injury. Collectively, these mechanisms contribute to the progressive death of neurons and long-term brain damage [3, 10]. Among these processes, neuroinflammation plays a pivotal role in exacerbating brain injury [11]. Microglia, the central nervous system’s (CNS) resident immune cells, are key mediators of this inflammatory response [12, 13]. Under normal conditions, microglia support neuronal survival and clear cellular debris. However, following ischemic injury, microglia become activated and adopt a pro-inflammatory phenotype, releasing cytokines and other mediators that exacerbate neuronal death. In particular, activated microglia initiate pyroptosis, a form of programmed cell death that further amplifies inflammation [14, 15]. A critical regulator of microglial activation is the NOD-like receptor family pyrin domain-containing 3 (NLRP3) inflammasome, a multiprotein complex that detects cellular stress and damage-associated molecular patterns (DAMPs) [16–18]. Once activated, the NLRP3 inflammasome promotes the maturation and release of pro-inflammatory cytokines, such as interleukin-1β (IL-1β) and interleukin-18 (IL-18), which exacerbate brain injury [16, 17]. Although inflammasome-mediated inflammation is essential for pathogen defense, its dysregulation in the CNS can result in excessive neuroinflammation and neuronal damage, as observed in HIBD. Thus, targeting the NLRP3 inflammasome to mitigate microglial activation and inhibit pyroptosis presents a promising therapeutic approach to reducing brain injury in neonates with HIBD. In recent years, mesenchymal stem cells (MSCs) have emerged as a potential therapy for ischemic brain injuries, including HIBD [19]. MSCs are multipotent cells capable of differentiating into various cell types and secreting bioactive molecules that promote tissue repair and modulate immune responses [20]. Recently, attention has shifted from direct MSC transplantation to the use of MSC-derived extracellular vesicles (EVs) [21]. EVs are small, membrane-bound particles secreted by cells, which facilitate intercellular communication by transferring proteins, lipids, and nucleic acids to target cells [22]. MSC-derived EVs are particularly appealing as a therapeutic tool because they retain the regenerative and immunomodulatory properties of MSCs without the risks associated with cell transplantation, such as tumorigenicity or immune rejection [23]. The human umbilical cord-derived MSC (hUC-MSC) is an especially promising source of EVs for therapeutic purposes. hUC-MSC-EVs carry a diverse range of bioactive molecules, including microRNAs (miRNAs), proteins, and lipids, which modulate key signaling pathways involved in inflammation, cell survival, and neuroprotection [24, 25]. Importantly, hUC-MSC-EVs can cross the blood–brain barrier, making them ideal candidates for treating neurological injuries like HIBD [24]. In preclinical models of ischemic brain injury, hUC-MSC-EVs have been shown to reduce inflammation, enhance neuronal survival, and promote neurogenesis, ultimately improving functional recovery [26, 27]. One potential mechanism through which hUC-MSC-EVs exert neuroprotective effects is by modulating the NLRP3 inflammasome [28]. By inhibiting NLRP3 activation, hUC-MSC-EVs can reduce microglial activation and pyroptosis, thereby dampening neuroinflammation [28]. Additionally, recent research suggests that hUC-MSC-EVs may promote the ubiquitination of NLRP3, a process critical for inhibiting its activation [29]. Protein kinase A (PKA) has been identified as a key regulator of NLRP3 ubiquitination, and it is hypothesized that hUC-MSC-EVs may enhance PKA activity to facilitate this process [30, 31]. This represents a novel and promising mechanism by which hUC-MSC-EVs may provide neuroprotection in the context of HIBD. Despite promising preclinical evidence, the precise mechanisms by which hUC-MSC-EVs regulate the NLRP3 inflammasome and provide neuroprotection in HIBD remain unclear [28, 32]. Understanding how hUC-MSC-EVs modulate inflammasome activation, microglial pyroptosis, and the downstream inflammatory response is critical to developing targeted therapies to mitigate brain injury in neonates [28]. Furthermore, the role of PKA-mediated ubiquitination of NLRP3 in the therapeutic effects [33] of hUC-MSC-EVs has yet to be fully elucidated and warrants further investigation [30, 34]. This study aims to explore the mechanisms through which hUC-MSC-EVs protect neonatal rats from HIBD, with a particular focus on their regulation of the NLRP3 inflammasome and PKA-mediated ubiquitination pathways.

## Materials and methods

### hUC-MSCs

This experiment aims to culture high-quality hUC-MSCs for extracting EVs to be used in subsequent therapeutic experiments. hUC-MSCs (Cyagen Biosciences, Guangzhou, China) were cultured in Dulbecco’s Modified Eagle’s Medium (DMEM) (A4192101, GIBCO, NY, USA), supplemented with 10% fetal bovine serum (FBS; 16000044, GIBCO), 100 µg/mL streptomycin, and 100 U/mL penicillin. Once the cell density reached 70%–80%, the cells were trypsinized and subcultured. Cells from passages 3–5 were utilized for the experiments that followed.

### Isolation and identification of hUC-MSC-EVs

To ensure the purity and quality of EVs used in the experiments, hUC-MSC-EVs were isolated using ultracentrifugation, and their morphology, size, and specific markers were verified [35]. To remove EVs from FBS, ultracentrifugation (120,000 × *g*, 3 h, 4 ^∘^C) was performed prior to adding FBS to DMEM. hUC-MSCs were then cultured in DMEM, with the cell suspension collected every other day for three consecutive days. The collected suspension was transferred to a conical tube and centrifuged (300 × *g*, 10 min, 4 ^∘^C) to precipitate cells, and the supernatant was retained. The supernatant was further centrifuged (16,500 ×*g*, 20 min, 4 ^∘^C) to remove cell debris, followed by filtration through a 0.22 µm strainer. The filtered supernatant was transferred to a new tube and subjected to ultracentrifugation (120,000 × *g*, 70 min, 4 ^∘^C) to isolate the EVs. After aspirating the supernatant, a second round of ultracentrifugation was performed under the same conditions. All ultracentrifugation steps were conducted using a benchtop centrifuge (Beckman Allegra X-15R) at 4 ^∘^C. The isolated EV particles were resuspended in 100 µL of sterile phosphate-buffered saline (PBS). The morphology of the EVs was observed using transmission electron microscopy (TEM) (Olympus, Tokyo, Japan), while their size and distribution were analyzed via nanoparticle tracking analysis (NTA). Western blotting was performed to assess the expression of positive EV markers (CD9, CD63, tumor susceptibility gene 101 [TSG101]) and the negative marker (Cis-Golgi matrix protein 130 [GM130]). As a control, hUC-MSCs were treated with 10 nM of the EV inhibitor GW4869 (Sigma-Aldrich, MO, USA) for 2 h [36]. PBS resuspension obtained from these treated hUC-MSCs, following the same EV isolation protocol, served as the control.

### Experimental animals

Specific pathogen-free Sprague-Dawley newborn rats were selected to ensure the reliability and consistency of the established HIBD model. These rats, aged seven days and weighing 12–16 g (half male and half female) [37], were sourced from the Medical Laboratory Animal Supply Base of Heilongjiang Province (Harbin, China). They were housed in a controlled animal facility with free access to food and water, maintained at a temperature of 22 ^∘^C–24 ^∘^C, a humidity level of 40%–70%, and a 12-h light/dark cycle.

### Establishment of HIBD models

HIBD was modeled by ligating the left common carotid artery and exposing the animals to hypoxia, simulating neonatal brain injury in humans. The HIBD model was established using a modified Rice–Vannucci method with specific pathogen-free Sprague-Dawley rats at postnatal day 7 (P7) [38–40]. Rats were selected based on inclusion criteria, including a body weight of 12–16 g and an age of exactly seven days (RRID: 131-11-001-B-000092). Animals with congenital anomalies, illness, or abnormal behavior were excluded from the study. The rats were anesthetized with 4% isoflurane for induction and 2% for maintenance. A midline neck incision was made to expose and permanently ligate the left common carotid artery using 5–0 silk sutures. The incision was then closed, and the pups were allowed to recover with their mothers for 1 h. Hypoxia was induced by placing the pups in a chamber maintained at 37 ^∘^C with a gas mixture of 8% oxygen and 92% nitrogen for 2.5 h. After hypoxia, the chamber was reoxygenated with ambient air for 10 min before the pups were returned to their mothers. The sham group underwent the same surgical procedure but without carotid artery ligation or hypoxia exposure. Postoperative care included 24-h monitoring for recovery, overall health, and thermoregulation, along with early neurological reflex (ENR) assessments. Behavioral testing was conducted after 10 weeks, followed by euthanasia using an overdose of sodium pentobarbital (100 mg/kg) [41].

### Grouping and treatment of newborn rats

Using the random number table method, newborn rats were divided into five groups (*n* ═ 18 per group): the sham group, the sham + EVs group, the HIBD group, the HIBD + EV-free supernatant (HIBD + EFS) group, and the HIBD + EVs group. Neonatal rats in the HIBD and HIBD + EFS groups received four intraperitoneal injections of 2 mL PBS or EFS in total [24, 41]. Similarly, rats in the HIBD + EFS and HIBD + EVs groups received four intraperitoneal injections of hUC-MSC-EVs (100 µg per injection) [24, 35]. The injections were administered as follows: the first 14 h before HIBD modeling, the second immediately prior to hypoxia exposure, the third after removal from the hypoxic chamber, and the fourth 3 h post-hypoxia. Twenty-four hours after modeling and early nerve reflex detection, six rats from each group were randomly selected for euthanasia using 100 mg/kg pentobarbital sodium, followed by TTC staining. The remaining 12 rats per group underwent the Morris water maze (MWM) test 10 weeks later [41]. After the MWM test, all rats were euthanized to collect hippocampal CA1 tissues. These rats were further divided as follows: six for hematoxylin and eosin (HE) staining, six for Terminal Deoxynucleotidyl Transferase dUTP Nick End Labeling (TUNEL) and immunofluorescence staining, and six for Western blotting and enzyme-linked immunosorbent assay (ELISA). All treatments, histological analyses, and functional assessments were conducted in a blinded and randomized manner.

### Detection of ENR

The ENR experiment aimed to evaluate the recovery of neurological function following HIBD by measuring reflexes, such as righting, cliff aversion, and geotaxis [42]. The specific assessment methods were as follows. Righting Reflex: Each rat was placed in a supine position, and the time (in seconds) taken to turn over to a normal prone position was recorded. Three trials were conducted for each rat, and the average time was calculated. Cliff-Aversion Reflex: This was tested by suspending each rat’s upper limbs over the edge of a board and recording the time it took to turn 90∘ away from the cliff edge. A maximum observation period of 20 s was set. If a rat did not turn 90∘ within this period, it was recorded as taking 20 s. Geotropic Reflex: The geotropic reflex was assessed by positioning each rat with its head downward on a 40∘ inclined board. The time required for the rat to turn around (rotating more than 90∘ to face upward) was recorded. As with the cliff-aversion test, a maximum observation time of 20 s was set, and rats that did not complete the movement within this period were recorded as taking 20 s.

### TTC staining

TTC staining was employed to quantify cerebral infarct volume (CIV) and evaluate the efficacy of hUC-MSC-EVs in mitigating brain damage after HIBD. The CIV was determined using TTC staining [41]. Twenty-four hours post-modeling, the ENR of all rats was assessed. Six rats from each group were randomly selected for euthanasia. Following euthanasia, the rats’ hearts were rapidly perfused with 100 mL of precooled saline (4 ^∘^C) to wash out the blood. The brains were quickly dissected on ice, with the cerebellum, olfactory bulb, and lower brainstem removed. The remaining brain tissue was preserved. The brains were frozen at -20 ^∘^C for 0.1 h, then sectioned into 2 mm-thick slices. These slices were incubated in 2% TTC solution (2530-85-0, Guidechem, Shanghai, China) in the dark at 37 ^∘^C for 0.5 h, flipping the slices midway to ensure uniform staining. After staining, the slices were fixed in 4% paraformaldehyde (PFA, Sigma-Aldrich) for 12 h. In TTC staining, normal brain tissue appeared deep red, while infarcted regions remained unstained. The percentage of CIV was calculated using ImageJ software (version 1.61; NIH Image, Bethesda, MD, USA) and was determined with the formula: percentage of CIV ═ (CIV/normal cerebral hemisphere volume) × 100% [41].

### MWM tests

MWM was employed to evaluate the spatial learning and memory of rats, analyzing the potential of hUC-MSC-EVs to improve cognitive function in HIBD rats. After 10 weeks of modeling, the MWM test was conducted to assess these cognitive parameters [43]. The setup included a circular stainless steel pool (120 cm in diameter, 60 cm in height) evenly divided into four quadrants. A hidden circular platform (10 cm in diameter) was submerged 1–2 cm below the water surface in the center of the target quadrant, with the water maintained at 23 ^∘^C–25 ^∘^C. A video tracking system (Mobile Benchmark, Shanghai, China) was installed above the pool. The experiment comprised two phases: a training phase (five days) and a test phase (one day). Prior to the trial, rats were allowed to explore the pool freely for 90 s without the platform to familiarize themselves with the environment. During the training phase, each rat was placed in one of the quadrants (excluding the platform quadrant) at 9 a.m. daily. The escape latency—the time taken to locate the platform—was recorded. If a rat successfully reached the platform within 90 s, it was allowed to remain on the platform for 20 s. Each test trial lasted 90 s. If a rat failed to find the platform within this time, the trial was stopped, and the rat was manually guided to the platform, where it stayed for 20 s to facilitate learning. In such cases, the escape latency was recorded as 90 s. Each rat underwent four training trials daily, with intervals of one hour between trials, and the average daily escape latency was calculated. Following each day’s training, the water in the maze was replaced with fresh water. On the sixth day, the space exploration test was conducted. The platform was removed, and rats were released from the quadrant opposite the previous platform location, allowing them to explore freely for 90 s. The escape latency and the number of times the rats crossed the previous platform location were recorded [44].

### HE staining

HE staining was performed to examine neuronal morphology in the hippocampal CA1 region and to assess the neuroprotective effects of hUC-MSC-EVs on HIBD rats. Following euthanasia, the rats underwent cardiac perfusion with 0.1 M PBS (pH 7.4). The hippocampal tissue was then immersed in 4% PFA at 4 ^∘^C for 48 h before undergoing a graded dehydration process: 70% ethanol for 3 min, 95% ethanol for 3 min, anhydrous ethanol twice (3 min and 2 min, respectively), and xylene twice (2 min and 3–5 min, respectively). The dehydrated tissue was subsequently embedded in paraffin. Paraffin-embedded tissues were sectioned into 2.5 µm-thick slices using a fully automated vibrating microtome (VT1000 S, Leica, Germany) and mounted onto poly-L-lysine-coated slides (Solarbio, Beijing, China). The sections were heated for 3 h and stored at room temperature until further analysis. HE staining was conducted using HE reagents (Solarbio) according to the manufacturer’s instructions. Neuronal morphology in the hippocampal CA1 region was then observed and imaged using an optical microscope (Olympus Corporation).

### Immunofluorescence

Immunofluorescence staining was performed to assess inflammation and neuronal survival in rat brain tissue, with the goal of investigating the anti-inflammatory and neuroprotective mechanisms of hUC-MSC-EVs. Rat brains were transcardially perfused with PBS, followed by 10 min of fixation with 4% PFA in PBS. The brains were then harvested, post-fixed in 4% PFA at 4 ^∘^C overnight, and cryoprotected in 30% sucrose for 48 h at 4 ^∘^C. Coronal brain sections (20 µm thick) were prepared using a cryostat (Leica CM1950, Leica Biosystems, Wetzlar, Germany) and stored at −20^∘^C until further use. Cultured microglia were fixed with 4% PFA in PBS for 20 min and permeabilized with 0.5% Triton X-100 for 15 min. Following this, blocking was performed using 2% bovine serum albumin (BSA). Tissue sections and cells were incubated overnight at 4 ^∘^C in a humidified chamber with primary antibodies, including rabbit anti-Tmem119 (1:500, GTX638642, GeneTex, CA, USA), anti-Iba1 (1:500, ab178846, Cambridge, MA, USA), and anti-CD68 rat monoclonal antibody (1:100, Serotec, Raleigh, NC, USA). The sections were then rinsed with 0.1 M PBS and incubated for 2 h with the appropriate secondary antibodies, including Alexa Fluor^®^ 488-conjugated goat anti-rabbit IgG H&L (1:200; Abcam, UK) and Cy3-Affinipure goat anti-rat IgG (1:100, Jackson ImmunoResearch, PA, USA). Nuclei were counterstained with 4’,6-diamidino-2-phenylindole (DAPI, Beyotime, Shanghai, China) for 5 min. Stained sections were observed using an Olympus BX53 microscope (Olympus, Center Valley, PA, USA). Quantitative colocalization analysis and overlap coefficient calculations (Manders’ coefficient) were conducted using ImageJ software. For each animal, three fields from the hippocampal CA1 region were randomly selected, and the number of double-positive cells was quantified using the Cell Counter plugin [45].

### Cell culture *in vitro*

**Figure S1.**
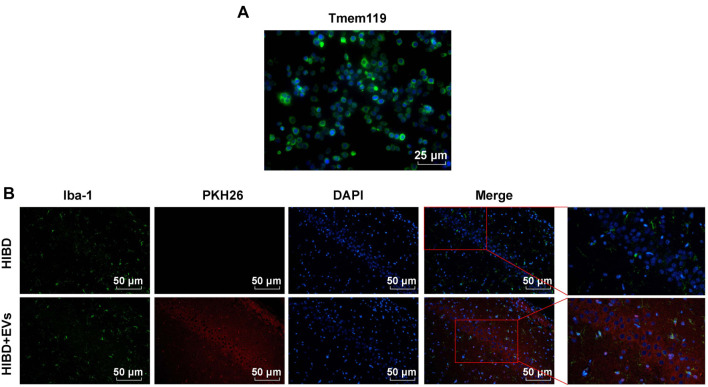
(A) The expression of microglia-specific marker Tmem119 was detected by immunofluorescence. Tmem119 labeled microglia (green); DAPI labeled nucleus (blue); (B) Uptake of PKH26-labeled hUC-MSC-EVs (red) by microglia *in vivo*. EVs were observed within Iba-1-positive microglia (green) in the hippocampal CA1 region; DAPI (blue) indicates nuclear staining. hUC-MSC-EV: Extracellular vesicles from human umbilical cord mesenchymal stem cells; EV: Extracellular vesicle; DAPI: 4’,6-diamidino-2-phenylindole.

Primary microglia and hippocampal neuronal cells were cultured *in vitro* to mimic *in vivo* hypoxic-ischemic conditions and evaluate the effects of hUC-MSC-EVs. Rat primary microglia, commercially sourced from SUNNCELL (SNP-R020, Wuhan, Hubei, China), were isolated via mechanical separation and maintained in rat microglia complete culture medium (SNPM-R020, SUNNCELL). Immunofluorescence analysis of the microglia-specific marker Tmem119 confirmed over 90% purity, high viability, and the absence of contaminants, such as bacteria, fungi, mycoplasma, or infectious viruses (Figure S1A). Hippocampal neuronal cells (H19-7; CRL-2526) were obtained from the Typical Culture Preservation Center (MD, USA) and cultured in DMEM (Invitrogen, Thermo Fisher Scientific, MA, USA) supplemented with 0.2 mg/mL G418, 0.001 mg/mL puromycin, and 10% FBS (Thermo Fisher). All cultures were incubated at 37 ^∘^C in a 5% CO_2_ and 95% air atmosphere, with the medium replaced every two days.

### Oxygen–glucose deprivation and reperfusion (OGD/R) model establishment and grouping

The OGD/R model was developed to simulate cerebral ischemia and assess the neuroprotective effects of hUC-MSC-EVs on nerve cells. As described previously [46], this model mimics an *in vivo* scenario of cerebral ischemia. Rat primary microglia were rinsed twice with PBS and incubated in glucose-free DMEM (without FBS) in an anoxic chamber (1% O_2_, 94% N_2_, 5% CO_2_, Thermo Fisher) at 37 ^∘^C. After 4 h of OGD exposure, the medium was replaced with standard medium supplemented with 10% FBS, and the cells were incubated under normoxic conditions for five days. Control cells were cultured in normal medium under normoxic conditions without undergoing OGD treatment. To ensure unbiased allocation, rat primary microglia were randomly assigned to six experimental groups using a random number generator. The groups were as follows. Control group: Maintained in normal medium under normoxic conditions. OGD/R group: Subjected to OGD/R treatment. OGD/R + EFS group: Treated with 0.1 µg/mL EFS for 1 h prior to OGD/R treatment. OGD/R + EVs group: Treated with 0.1 µg/mL hUC-MSC-EVs for 1 h [26] prior to OGD/R treatment. OGD/R + EVs + N group: Treated with 20 µM Nigericin and 0.1 µg/mL hUC-MSC-EVs for 1 h [47, 48] prior to OGD/R treatment. OGD/R + EVs + H89 group: Treated with 10 µM H89 [49, 50] and 0.1 µg/mL hUC-MSC-EVs for 1 h prior to OGD/R treatment. Nigericin, an activator of the NLRP3 inflammasome (Thermo Fisher), and H89, a selective inhibitor of PKA kinase (Cayman Chemical Company, MI, USA), were used to modulate specific cellular pathways. For the H19-7 neuronal cell line, cells were randomly divided into five groups using a random number generator. CM-C group: Cultured in conditioned medium (CM) derived from the control group’s rat primary microglia. CM-O group: Cultured in CM from the OGD/R group. CM-O-EFS group: Cultured in CM from the OGD/R + EFS group. CM-O-EVs group: Cultured in CM from the OGD/R + EVs group. CM-O-EVs-N group: Cultured in CM from the OGD/R + EVs + N group. Randomization of both microglia and H19-7 cells was performed in each experimental batch to minimize bias. Additionally, all experiments were conducted in a blinded manner to ensure objective analysis of results.

### Uptake test

EVs were labeled with PKH26 dye to evaluate their uptake by cells *in vitro* and assess the efficiency of hUC-MSC-EV internalization. Specifically, 1 mL of PKH26 dye solution (1:1000; Sigma-Aldrich [41]) was mixed with EVs (20 µg protein) for 20 min, followed by washing with PBS and centrifugation at 100,000 × *g* for 70 min. Rat primary microglia and H19-7 cells (3 × 10^ImEquation4^ cells/well) were seeded in 24-well plates. Some cells were incubated at 37 ^∘^C with 5% CO_2_ under normoxic conditions, while others were incubated in an anoxic chamber (1% O_2_, 94% N_2_, 5% CO_2_). After 48 h, prestained EVs (80 µg/mL) were added and cultured for one day. EV uptake was analyzed using a confocal fluorescence microscope (Carl Zeiss, Oberkochen, Germany).

### Western blotting

Western blotting was employed to detect the expression of proteins related to inflammation and apoptosis, evaluating the effects of hUC-MSC-EVs on these processes in HIBD. Cells, EVs, or brain tissues were lysed using radioimmunoprecipitation assay (RIPA) buffer (Beyotime) supplemented with complete TM protease inhibitors (11836145001, Roche, Basel, Switzerland) at a working concentration of one tablet per 50 mL of extract. After collecting the supernatant, protein content was quantified using bicinchoninic acid (BCA) protein quantification kits (Boster Biological Technology, Hubei, China). The isolated proteins were separated using 10% sodium dodecyl sulfate-polyacrylamide gel electrophoresis (SDS-PAGE) and subsequently transferred onto polyvinylidene fluoride (PVDF) membranes. Membranes were blocked for 1 h with 5% skim milk in Tris-buffered saline containing 0.1% Tween-20 (TBST, 20 mM Tris, 137 mM NaCl, 0.1% Tween-20) and incubated overnight (12 h) at 4 ^∘^C with the following primary antibodies: CD63 (1:1000, ab134045, Abcam, Cambridge, UK), TSG101 (1:1000, ab30871, Abcam), GM130 (1:1000, ab52649, Abcam), CD9 (1:1000, ab236630, Abcam), NLRP3 (1:1000, ab263899, Abcam), apoptosis-associated spot-like protein (ASC) (1:1000, ab283684, Abcam), cleaved-caspase-1 (1:1000, 4199, Cell Signaling Technology), CD68 (1:1000, ab213363, Abcam), N-terminal cleaved gasdermin-D (GSDMD-N) (1:1000, ab215203, Abcam), and β-actin (1:2000, ab8227, Abcam). After washing with TBST (Solarbio), membranes were incubated with goat anti-rabbit HRP-labeled secondary antibody (1:2000, AB6721, Abcam) for 2 h. Protein bands were visualized using an enhanced chemiluminescence (ECL) working solution (EMD Millipore, MA, USA). ImageJ software was used to quantify the pixel density of each Western blotting band, with β-actin serving as an internal reference [51].

### TUNEL assay

The TUNEL assay was used to detect cell apoptosis in the brain tissue of HIBD rats, assessing the potential of hUC-MSC-EVs to inhibit neuronal apoptosis [52]. Cell death was identified using Click-iT™ Plus TUNEL kits (Thermo Fisher), following the manufacturer’s instructions precisely. Nuclei were counterstained with DAPI (Beyotime). Fluorescent images were captured using a fluorescence microscope (Leica Microsystems, Wetzlar, Germany), where TUNEL-positive cells exhibited green fluorescence, and nuclei appeared blue. ImageJ software (version 1.61; NIH Image), along with the Cell Counter plugin, was employed to quantify the TUNEL-positive cells in the vulnerable hippocampal CA1 region.

### ELISA

ELISA was employed to measure the levels of the inflammatory cytokines IL-1β and IL-18, assessing the regulatory effects of hUC-MSC-EVs on inflammation. Total protein concentration was determined using BCA assay kits (Beyotime). The protein levels of IL-1β and IL-18 were quantified using specific ELISA kits for IL-1β (R&D Systems, MN, USA) and IL-18 (R&D Systems) following the manufacturer’s protocols. Data were collected with a microplate reader (Bio-Rad 680, Bio-Rad, CA, USA).

### 3-(4,5-dimethylthiazol-2-yl)-2,5-diphenyltetrazolium bromide (MTT) assay

The MTT assay was utilized to assess cell viability and to examine the effects of hUC-MSC-EVs on the survival of cultured nerve cells [53]. Cell viability was determined using MTT kits (M1020, Solarbio), with optical density (OD) values measured using a microplate reader at a wavelength of 450 nm. All procedures strictly followed the instructions provided in the kit manual.

### Flow cytometry analysis

Flow cytometry was used to analyze microglial activation in brain tissues. Single-cell suspensions were prepared by enzymatically digesting tissues with collagenase IV (0.5 mg/mL, Sigma-Aldrich) and DNase I (0.1 mg/mL, Roche), followed by filtration through a 70 µm cell strainer. Cells were stained with anti-CD45-FITC (1:200, ab317446, Abcam), anti-CD11b-PE (1:200, ab8878, Abcam), and anti-CD68-APC (1:200, ab283654, Abcam) antibodies at 4 ^∘^C for 30 min in the dark. To evaluate microglial activation, data acquisition was performed on a BD FACSCanto II flow cytometer, with 1 × 10^6^ events collected per sample. The gating strategy involved first selecting live single cells based on forward scatter (FSC) and side scatter (SSC) to exclude debris and doublets. Microglia were identified as CD11bˆ+ cells, a common marker for brain-resident microglia, and activated microglia were further defined as CD11bˆ+CD68ˆ+ cells, with CD68 serving as a marker for activation. Data analysis was conducted using FlowJo software (version 10.6.2). The proportion of activated microglia (CD11bˆ+CD68ˆ+) was quantified relative to the total microglial population (CD11bˆ+) [54].

### Co-immunoprecipitation (Co-IP) analysis

Co-IP was performed to detect the ubiquitination of NLRP3 and to investigate whether hUC-MSC-EVs inhibit inflammasome activation by promoting NLRP3 ubiquitination [30]. Cell lysates were prepared by adding lysis buffer [50 mM Tris-HCl, 5 mM EDTA, 150 mM NaCl, 0.5% (vol/vol) Nonidet-P40, and 10% (vol/vol) glycerol, pH 7.4] supplemented with complete protease and phosphatase inhibitor mixtures (50X, P1049, Beyotime). The lysates were incubated with Protein A/G agarose beads (Bimake, TX, USA) and an anti-NLRP3 antibody (1:200, AG-20B-0014, AdipoGen, CA, USA) at 4 ^∘^C for 12 h. After incubation, the beads were washed five times with cold IP buffer and boiled in 1× SDS-PAGE sample loading buffer for 8 min. The eluted proteins were analyzed via Western blotting using an anti-NLRP3 antibody (1:1000, AG-20B-0014, AdipoGen) and an antibody specifically recognizing the K63-linked polyubiquitin chain (1:1000, ab179434, Abcam).

### Measurement of PKA kinase activity

PKA kinase activity was measured to determine whether hUC-MSC-EVs regulate NLRP3 ubiquitination via activation of the PKA signaling pathway, thereby elucidating their anti-inflammatory mechanism [55]. Samples were homogenized in a lysis buffer containing 0.4 mmol/L 3-isobutyl-methylxanthine (IBMX), a phosphodiesterase inhibitor (Sigma-Aldrich). Basal PKA activity and maximum PKA activity (induced by 1 µM cAMP) in the homogenate were evaluated using PKA kinase activity assay kits (Assay Designs, MI, USA) following the manufacturer’s protocol. In summary, 40 µL of sample or recombinant PKA standards (at various concentrations) were added to the assay plate wells, followed by the addition of 10 µL recombinant ATP to each well. The plate was incubated at 30 ^∘^C for 1.5 h. After incubation, the reagents were aspirated, and the plates were washed four times with 300 µL wash buffer, then dried with an absorbent towel. For antigen–antibody binding, 25 µL of rabbit anti-phospho-PKA substrate antibody (1:1000, 9624, Cell Signaling Technology) and 25 µL of goat anti-rabbit IgG were added to each well. The plates were sealed and shaken during a 1-h incubation. Subsequently, the wells were aspirated and washed four times with wash buffer. Next, 100 µL of 3,3’,5,5’-tetramethylbenzidine substrate solution was added and incubated for 30 min, followed by the addition of 50 µL termination solution. OD was measured at 450 nm using a microplate reader. PKA activity was calculated using linear regression from standard curves and normalized to protein concentration.

### Ethical statement

All experimental protocols were reviewed and approved by the Research and Ethics Committee of The Second Affiliated Hospital of Harbin Medical University (approved number: YJSDW-2022-035). All procedures conformed to internationally accepted guidelines and ethics for animal research. Great effort was made to reduce the total number of animals used and to minimize their suffering.

### Statistical analysis

GraphPad Prism 8.01 (GraphPad Software Inc., CA, USA) was utilized for statistical analysis and data plotting. The outlier test was carried out by the Grubbs method, and no data points were excluded. Measurement data were presented as the mean ± standard deviation (SD). The *t*-test was utilized for data comparison between two groups, one-way analysis of variance (ANOVA) was adopted to compare multiple groups, followed by Tukey’s test. The *P* value was calculated using a two-tailed test, where the value of *P* < 0.05 was indicative of a statistically significant difference.

**Figure 1.**
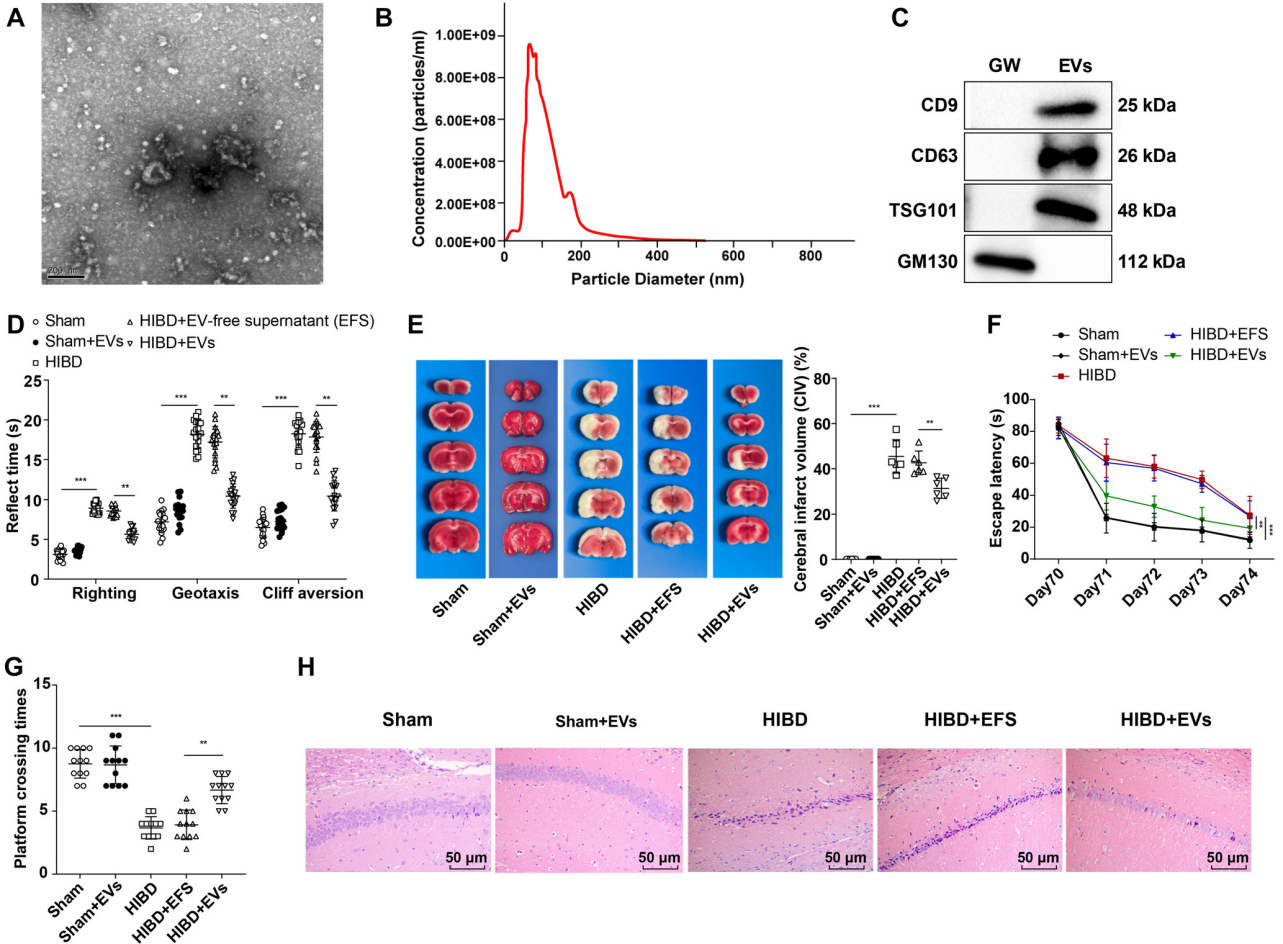
**hUC-MSC-EVs attenuated brain damage in HIBD neonatal rats.** (A) The morphology of hUC-MSC-EVs was observed using TEM; (B) The particle size of hUC-MSC-EVs was measured using NTA; (C) Western blotting was used to detect the expression of hUC-MSC-EVs surface markers TSG101, CD63, CD9, and GM130, and GW4869 was used as negative control; (D) Detection of ENR (*n* ═ 18); (E) TTC staining to detect the infarct area and the percentage of infarct volume (*n* ═ 6), infarct area: Gray; non-infarcted area: Bright red; Morris water maze test (*n* ═ 12); (F) The escape latency; (G) Number of platform crossings; (H) HE staining to observe the damage of nerve cells in hippocampal CA1 region *n* ═ 6. Data were expressed as mean ± SD. One-way ANOVA analysis was performed among multiple groups, and Tukey’s test was used for the post-hoc test. **P < 0.01, ***P < 0.001. hUC-MSC-EV: Extracellular vesicles from human umbilical cord mesenchymal stem cells; SD: Standard deviation; HIBD: Hypoxic-ischemic brain injury; ANOVA: Analysis of variance; NTA: Nanoparticle tracking analysis; ENR: Early neurological reflex; TEM: Transmission electron microscopy; HE: Hematoxylin and eosin.

## Results

### hUC-MSC-EVs attenuated HIBD in neonatal rats

To investigate whether hUC-MSC-EVs could attenuate HIBD in neonatal rats, we first cultured hUC-MSCs *in vitro* and isolated EVs. Morphological observation using TEM revealed the typical cup-shaped structure of EVs (Figure 1A). NTA analysis showed that the diameter of EVs ranged from 40 to 150 nm, with a particle concentration of 9.23 × 10^8^/mL (Figure 1B). Western blotting analysis confirmed the significant expression of EV markers CD9, CD63, and TSG101 in the EVs group compared to the GW group, while GM130, a negative control marker, was not expressed (Figure 1C). These results confirmed the successful isolation of hUC-MSC-EVs. Additionally, immunofluorescence experiments using PKH26 and Iba-1 double staining verified that microglia had phagocytosed EVs, demonstrating that intraperitoneally injected EVs crossed the blood–brain barrier, entered the hippocampus, and were taken up by microglia (Figure S1B). These findings provide strong evidence for the involvement of EVs in HIBD. Next, we established neonatal rat HIBD models and treated HIBD rats with hUC-MSC-EVs. After 10 weeks, the ENR test showed that HIBD rats exhibited significantly prolonged cliff aversion, geotaxis, and righting reflexes. Treatment with hUC-MSC-EVs markedly improved all three reflexes (Figure 1D, all *P* < 0.01). TTC staining further confirmed that the CIV in the HIBD group (45.4% ± 7.4%) was significantly higher than in the Sham group (0% ± 0%). Meanwhile, the CIV in the HIBD + EVs group (31.4% ± 4.4%) was markedly lower than in the HIBD + EFS group (42.6% ± 5.2%) (Figure 1E, all *P* < 0.01). To evaluate learning and memory abilities, rats underwent MWM tests on days 70–74. Rats in the HIBD group showed a significantly increased escape latency and reduced platform crossing frequency compared to the Sham group. However, rats in the HIBD + EVs group demonstrated shorter escape latency and increased platform crossing frequency compared to the HIBD + EFS group (Figure 1F and 1G, all *P* < 0.01). HE staining results revealed that nerve cells in the hippocampal CA1 region of the Sham group were intact and neatly arranged, with round or oval nuclei centrally located, less chromatin, and visible nucleoli. In contrast, the number of surviving neurons in the hippocampal CA1 region of the HIBD group was significantly reduced. Notably, the HIBD + EVs group showed a substantial increase in viable neurons compared to the HIBD + EFS group (Figure 1H, all *P* < 0.01). This study successfully isolated and identified hUC-MSC-EVs. *In vivo* experiments demonstrated that hUC-MSC-EVs significantly improved neurological reflexes, reduced CIV, enhanced learning and memory performance, and increased the number of surviving neurons in the hippocampal CA1 region of HIBD rats, highlighting their neuroprotective effects. Across all experiments, no significant differences were observed between the Sham + EVs and Sham groups, confirming that hUC-MSC-EVs do not affect baseline physiological or neurological parameters in healthy control rats (Figure 1A–1H, all *P* > 0.05). This lack of significant changes in the Sham + EVs group further reinforces that the beneficial effects of hUC-MSC-EVs are specific to the HIBD condition.

### hUC-MSC-EVs inhibited NLRP3 inflammasome activation to restrain microglial activation and reduce pyroptosis in HIBD rats

**Figure 2.**
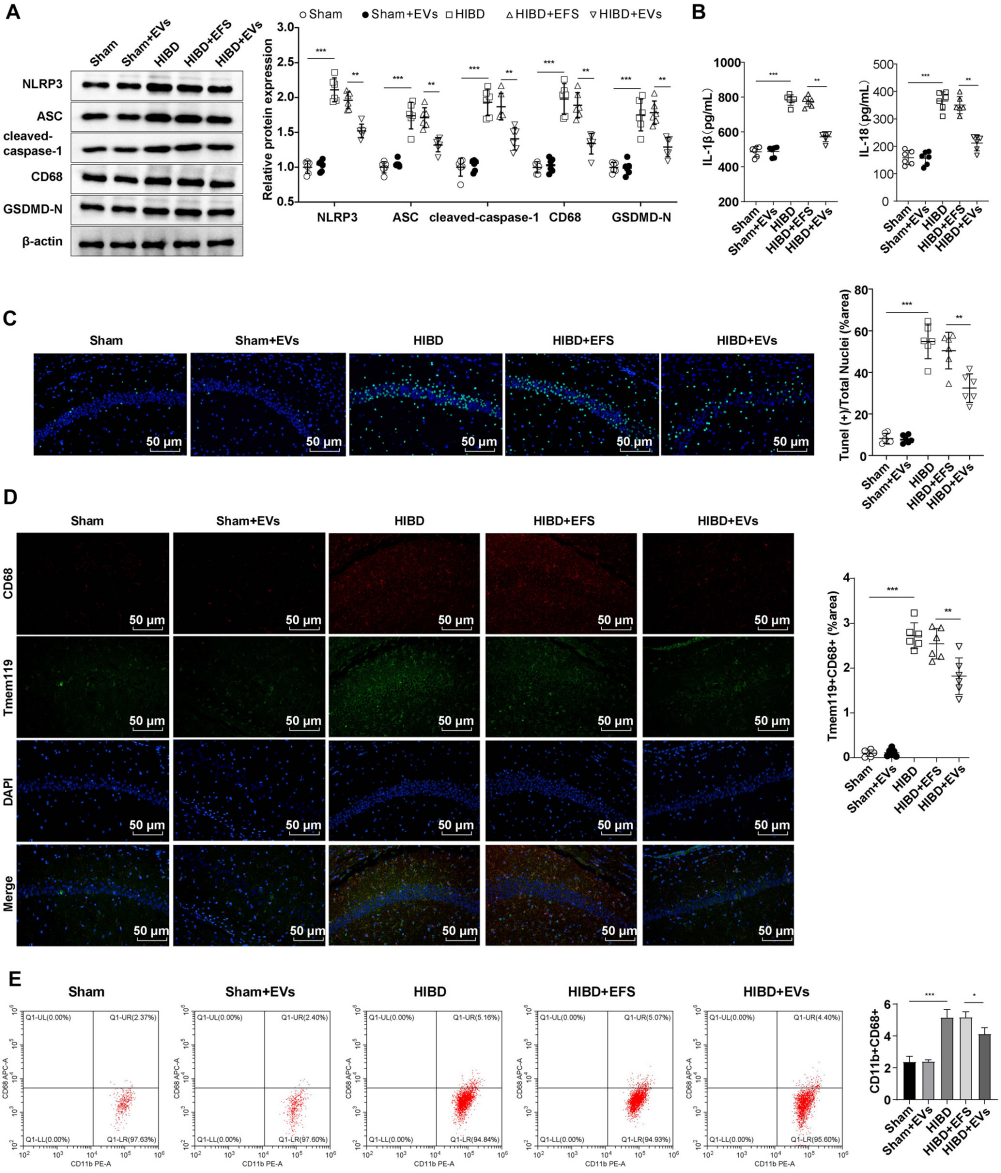
**hUC-MSC-EVs inhibited NLRP3 activation to inhibit microglia activation and reduce pyroptosis in HIBD rats.** (A) The levels of NLRP3, ASC, cleaved caspase-1, CD68, and GSDMD-N were detected using Western blotting; (B) The levels of inflammatory cytokines IL-18 and IL-1β were detected using ELISA; (C) The number of cell deaths in the hippocampus was detected by TUNEL staining. TUNEL positive: Green; DAPI: Blue; (D) The levels of Tmem119 and CD68 in the hippocampal CA1 region were detected using immunofluorescence. Tmem119: Green. CD68: Red; DAPI: Blue. Microglia activation was represented by Tmem119+CD68+; (E) Flow cytometry analysis of CD68+ microglia populations. Data were expressed as mean ± SD (*n* ═ 6). One-way ANOVA analysis was performed among multiple groups, and Tukey’s test was used for the post-hoc test. **P < 0.01, ***P < 0.001. hUC-MSC-EV: Extracellular vesicles from human umbilical cord mesenchymal stem cells; NLRP3: NOD-like receptor family pyrin domain-containing 3; SD: Standard deviation; IL-18: Interleukin-18; IL-1β: Interleukin-1β; TUNEL: Terminal deoxynucleotidyl transferase dUTP Nick end labeling; ELISA: Enzyme-linked immunosorbent assay; GSDMD-N: N-terminal cleaved gasdermin-D; HIBD: Hypoxic-ischemic brain injury; ANOVA: Analysis of variance; DAPI: 4’,6-diamidino-2-phenylindole.

A recent study highlighted the critical role of the NLRP3 inflammasome in microglial activation [56]. It is speculated that the protective effects of hUC-MSC-EVs on HIBD may stem from their ability to inhibit NLRP3 inflammasome activation, thereby suppressing microglial activation and reducing pyroptosis. To investigate this, Western blotting was used to measure the levels of NLRP3 inflammasome-related proteins (NLRP3, cleaved caspase-1, and ASC), a microglial activation marker (CD68), and a pyroptosis-related protein (GSDMD-N). Following hypoxic-ischemic induction, the levels of NLRP3, ASC, cleaved caspase-1, CD68, and GSDMD-N in rat brain tissue were significantly upregulated but were notably decreased after hUC-MSC-EVs treatment compared to the HIBD + EFS group (Figure 2A, all *P* < 0.01). ELISA results revealed that rats in the HIBD + EVs group exhibited lower levels of inflammatory cytokines IL-1β and IL-18 than those in the HIBD and HIBD + EFS groups (Figure 2B, all *P* < 0.01). TUNEL staining further demonstrated that the number of dead cells (green fluorescence) in the hippocampal CA1 region was significantly increased in the HIBD group, while the HIBD + EVs group displayed a reduced number of dead cells compared to the HIBD + EFS group (Figure 2C, all *P* < 0.01). Immunofluorescence analysis of Tmem119 and CD68 levels in the hippocampal CA1 region showed a higher proportion of Tmem119+CD68+ positive cells in the HIBD group, which was markedly reduced in the HIBD + EVs group relative to the HIBD + EFS group (Figure 2D, all *P* 0.01). Additionally, flow cytometry revealed elevated levels of CD68+ activated microglia in the HIBD group, which were significantly decreased following hUC-MSC-EVs treatment in the HIBD + EVs group (Figure 2E, all *P* < 0.01). These findings indicate that hUC-MSC-EVs effectively inhibit NLRP3 inflammasome activation, thereby reducing microglial activation and pyroptosis. Moreover, hUC-MSC-EVs decreased inflammatory cytokine levels (IL-1β and IL-18) and cell death in the hippocampal CA1 region, confirming their anti-inflammatory and neuroprotective properties. Notably, the absence of significant changes in the Sham + EVs group further supports the specificity of hUC-MSC-EVs’ neuroprotective effects in the HIBD condition (Figure 2A–2E, all *P* > 0.05).

### hUC-MSC-EVs prevented OGD/R-induced rat primary microglia activation and pyroptosis by blocking NLRP3 inflammasome activation

**Figure 3.**
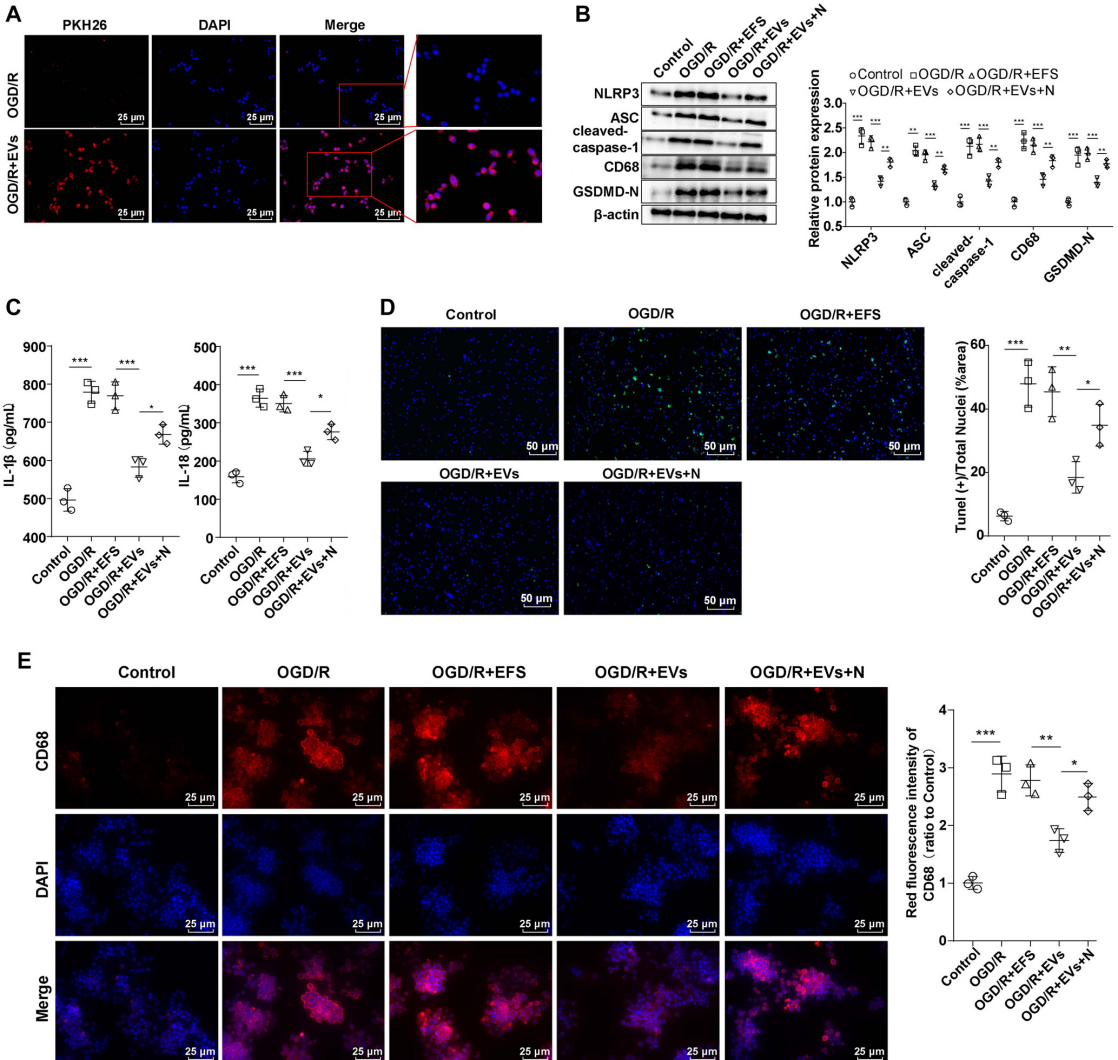
**Inhibition of NLRP3 activation by hUC-MSC-EVs inhibited pyroptosis of OGD/R-induced rat microglia.** The cells were treated with 0.1 µg/mL EFS, hUC-MSC-EVs, 20 µM Nigericin and 0.1 µg/mL hUC-MSC-EVs for 1 h before OGD/R treatment. (A) The uptake of hUC-MSC-EVs by rat microglia was observed by PKH26 fluorescence staining. hUC-MSC-EV staining: Red; DAPI staining: Blue; (B) The levels of NLRP3, ASC, cleaved caspase-1, CD68, and GSDMD-N were detected using Western blotting; (C) The levels of inflammatory cytokines IL-18 and IL-1β were detected using ELISA; (D) The number of dead cells in the hippocampus was detected by TUNEL staining. TUNEL positive: Green; DAPI: Blue; (E) The level of CD68 was detected by immunofluorescence. CD68: Red; DAPI: Blue. Cell experiments were repeated three times independently. Data were expressed as mean ± SD. *P < 0.05, **P < 0.01, ***P < 0.001. hUC-MSC-EV: Extracellular vesicles from human umbilical cord mesenchymal stem cells; NLRP3: NOD-like receptor family pyrin domain-containing 3; SD: Standard deviation; IL-18: Interleukin-18; IL-1β: Interleukin-1β; TUNEL: Terminal deoxynucleotidyl transferase dUTP Nick end labeling; ELISA: Enzyme-linked immunosorbent assay; GSDMD-N: N-terminal cleaved gasdermin-D; OGD/R: Oxygen–glucose deprivation/reoxygenation; DAPI: 4’,6-diamidino-2-phenylindole; EFS: EV-free supernatant.

hUC-MSC-EVs may block the activation of hippocampal microglia in the CA1 region of the hippocampus by inhibiting NLRP3 inflammasome activation and reducing pyroptosis in HIBD rats, thereby alleviating HIBD. To investigate this, we purchased rat primary microglia with over 90% purity and no contamination (Figure S1A). These cells were treated with hUC-MSC-EVs and Nigericin (an NLRP3 inflammasome activator), followed by the establishment of OGD/R models. The uptake assay confirmed that hUC-MSC-EVs were internalized by rat primary microglia (Figure 3A). Western blotting analysis revealed that the OGD/R group exhibited elevated levels of NLRP3, ASC, cleaved-caspase-1, CD68, and GSDMD-N. These levels were significantly reduced after treatment with hUC-MSC-EVs compared to the OGD/R + EFS group, but they increased in the OGD/R + EVs + N group (Figure 3B, all *P* < 0.01). Similarly, ELISA results showed that IL-1β and IL-18 levels were significantly upregulated in the OGD/R group. Treatment with hUC-MSC-EVs reduced these levels relative to the OGD/R + EFS group, but their levels were higher in the OGD/R + EVs + N group compared to the OGD/R + EVs group (Figure 3C, all *P* < 0.05). TUNEL staining indicated a marked increase in cell death (green fluorescence) in the OGD/R group. However, treatment with hUC-MSC-EVs significantly reduced cell death compared to the OGD/R + EFS group. Nigericin combined with hUC-MSC-EVs caused more cell death than hUC-MSC-EVs alone (Figure 3D, both *P* < 0.05). Immunofluorescence analysis revealed that the OGD/R group displayed dramatically higher CD68 fluorescence intensity compared to the Control group. Conversely, the OGD/R + EVs group had significantly lower CD68 fluorescence intensity than the OGD/R + EFS group (Figure 3E, all *P* < 0.05). In summary, hUC-MSC-EVs suppressed OGD/R-induced rat primary microglial activation and pyroptosis by inhibiting NLRP3 inflammasome activation. *In vitro* experiments further demonstrated that hUC-MSC-EVs were internalized by primary microglia and effectively prevented OGD/R-induced pyroptosis and microglial activation, supporting their neuroprotective effects.

### hUC-MSC-EVs can alleviate the pyroptosis of OGD/R-exposed rat primary microglia to neurons

**Figure 4.**
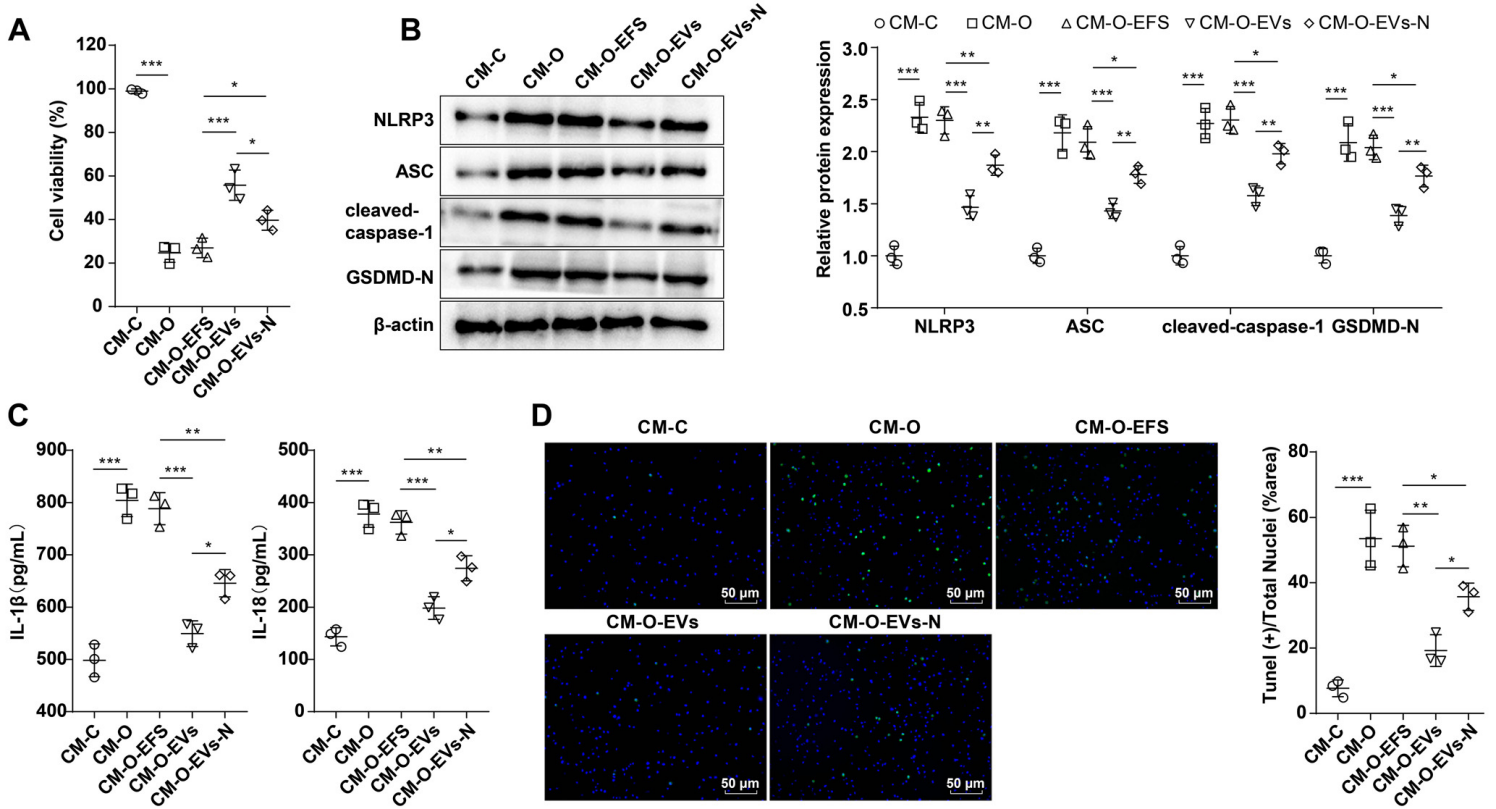
**hUC-MSC-EVs attenuated neuronal pyroptosis in OGD/R-exposed rat microglia.** (A) Cell viability was determined by MTT assay; (B) The levels of NLRP3, ASC, cleaved caspase-1, and GSDMD-N were detected using Western blotting; (C) The levels of inflammatory cytokines IL-18 and IL-1β were detected using ELISA; (D) The number of dead cells in the hippocampus was detected by TUNEL staining. TUNEL positive: Green; DAPI: Blue. Cell experiments were repeated three times independently. Data were expressed as mean ± SD. *P < 0.05, **P < 0.01, ***P < 0.001. hUC-MSC-EV: Extracellular vesicles from human umbilical cord mesenchymal stem cells; NLRP3: NOD-like receptor family pyrin domain-containing 3; SD: Standard deviation; IL-18: Interleukin-18; IL-1β: Interleukin-1β; TUNEL: Terminal deoxynucleotidyl transferase dUTP Nick end labeling; ELISA: Enzyme-linked immunosorbent assay; GSDMD-N: N-terminal cleaved gasdermin-D; OGD/R: Oxygen–glucose deprivation/reoxygenation; DAPI: 4’,6-diamidino-2-phenylindole; MTT: 3-(4,5-dimethylthiazol-2-yl)-2,5-diphenyltetrazolium bromide.

To investigate the impact of OGD/R-exposed rat primary microglia on neurons, H19-7 cells were cultured using the CM derived from rat primary microglia of each experimental group. An MTT assay revealed that cell viability in the CM-O group was significantly reduced compared to the CM-C group. However, cell viability was notably increased in the CM-O-EVs group compared to the CM-O-EFS group. Conversely, H19-7 cells exhibited lower viability in the CM-O-EVs-N group than in the CM-O-EVs group (Figure 4A, all *P* < 0.05). Western blotting analysis showed elevated levels of NLRP3, ASC, cleaved caspase-1, and GSDMD-N proteins in the CM-O group relative to the CM-C group. These levels were markedly reduced in the CM-O-EVs group compared to the CM-O-EFS group, but higher levels were observed in the CM-O-EVs-N group (Figure 4B, all *P* < 0.01). ELISA results demonstrated that the CM-O group exhibited significantly higher levels of IL-1β and IL-18 compared to the CM-C group. However, these levels decreased in the CM-O-EVs group compared to the CM-O-EFS group but increased in the CM-O-EVs-N group (Figure 4C, all *P* 0.05). TUNEL staining revealed a higher number of cell deaths (green fluorescence) in the CM-O group compared to the CM-C group. In contrast, the CM-O-EVs group showed reduced cell death compared to the CM-O-EFS group. Interestingly, Nigericin combined with hUC-MSC-EVs treatment significantly increased cell death in H19-7 cells compared to hUC-MSC-EVs alone (Figure 4D, all *P* < 0.05). Moreover, the CM-O-EVs-N group showed notable reductions in the expression of NLRP3, ASC, cleaved caspase-1, GSDMD-N, IL-18, and IL-1β, as well as in the number of cell deaths. These changes were accompanied by a significant increase in cell viability compared to the CM-O-EFS group (Figure 4A–4D, all *P* < 0.05). These findings suggest that the protective effects of EVs were not completely abolished by NLRP3 activation. Co-culture experiments using OGD/R-activated microglia further demonstrated that hUC-MSC-EVs significantly enhanced neuronal viability, reduced pyroptosis marker expression, and decreased inflammatory cytokine levels. These results highlight the ability of hUC-MSC-EVs to mitigate the harmful effects of microglial activation on neurons.

### hUC-MSC-EVs promoted NLRP3 ubiquitination through activation of PKA kinase

**Figure 5.**
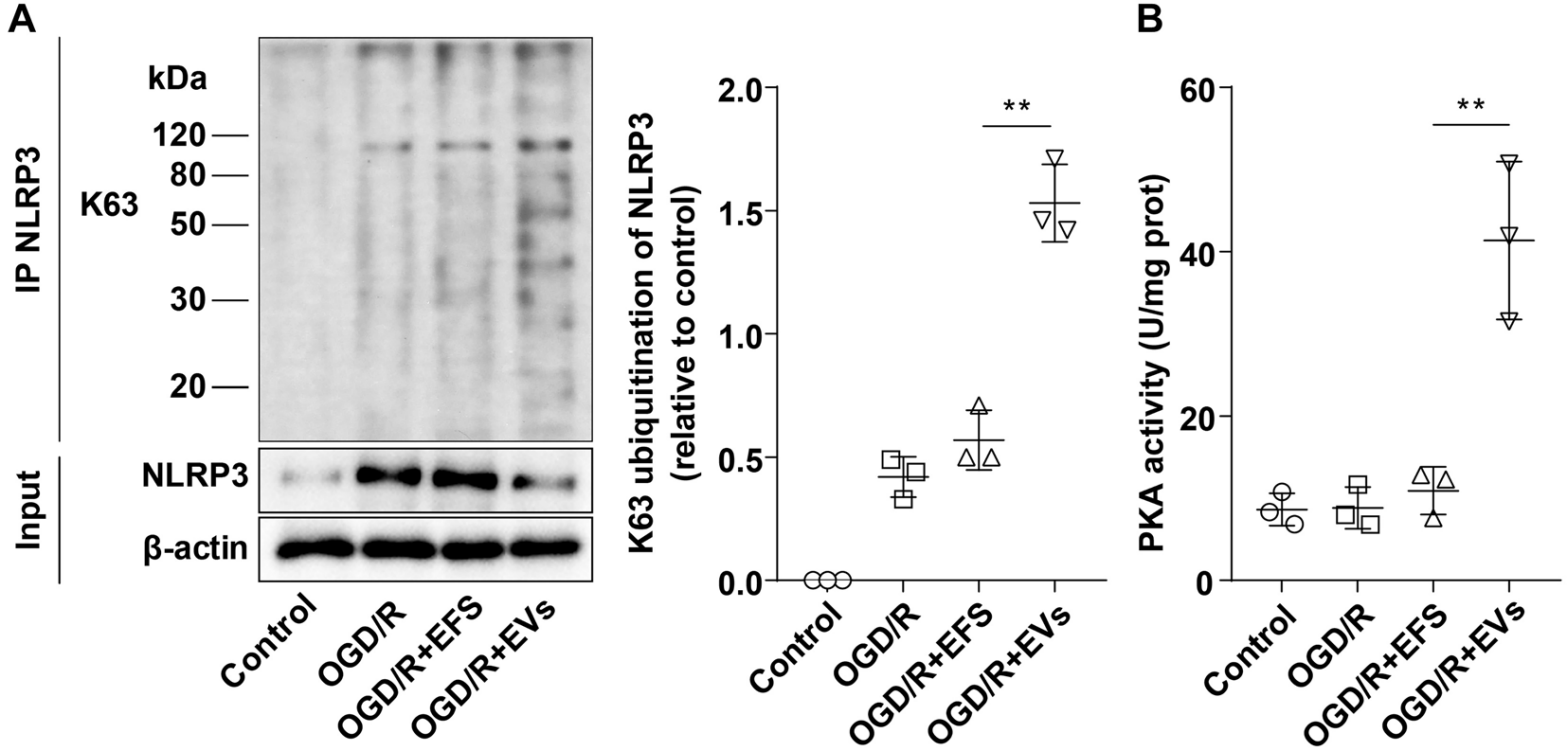
**hUC-MSC-EVs inhibited NLRP3 inflammasome activation by promoting NLRP3 ubiquitination through activation of PKA kinase.** (A) The K63-linked polyubiquitination level of NLRP3 was detected by Co-IP assay, and K63-linked polyubiquitination blocked NLRP3 activation. Input: Positive control; IP NLRP3: K63 was immunoprecipitated with the NLRP3 antibody to detect the interaction between the two; (B) PKA kinase activity was detected by the kits. Cell experiments were repeated three times independently. Data were expressed as mean ± SD. **P < 0.01. hUC-MSC-EV: Extracellular vesicles from human umbilical cord mesenchymal stem cells; NLRP3: NOD-like receptor family pyrin domain-containing 3; PKA: Protein kinase A; SD: Standard deviation; Co-IP: Co-immunoprecipitation.

Maresin1-induced PKA kinase activation promotes K63-linked ubiquitination of NLRP3 [30], which, in turn, inhibits NLRP3 activation [57]. Based on this, we hypothesized that hUC-MSC-EVs suppress NLRP3 inflammasome activation by enhancing NLRP3 ubiquitination via PKA kinase activation. To test this, we measured the K63-linked polyubiquitination level of NLRP3 using a Co-IP assay. No K63-linked polyubiquitination of NLRP3 was detected in the control group, while it was observed in both the OGD/R and OGD/R + EFS groups with no significant differences between them. Notably, the K63-linked polyubiquitination level of NLRP3 was significantly higher in the hUC-MSC-EV-treated group compared to the OGD/R + EFS group, demonstrating that NLRP3 activation was effectively inhibited (Figure 5A, all *P* < 0.01). Further, PKA activity assays revealed low PKA activity in both the control and OGD/R groups. In contrast, the OGD/R + EVs group exhibited significantly higher PKA activity compared to the OGD/R + EFS group, indicating that hUC-MSC-EVs can activate PKA kinase (Figure 5B, *P* < 0.01). These findings collectively show that hUC-MSC-EVs promote K63-linked ubiquitination of NLRP3 through PKA kinase activation, thereby suppressing NLRP3 inflammasome activation. This mechanism highlights the anti-inflammatory pathway mediated by hUC-MSC-EVs.

### Inhibition of PKA kinase activity partially reversed the ameliorative effect of hUC-MSC-EVs on OGD/R-induced pyroptosis of rat primary microglia

**Figure 6.**
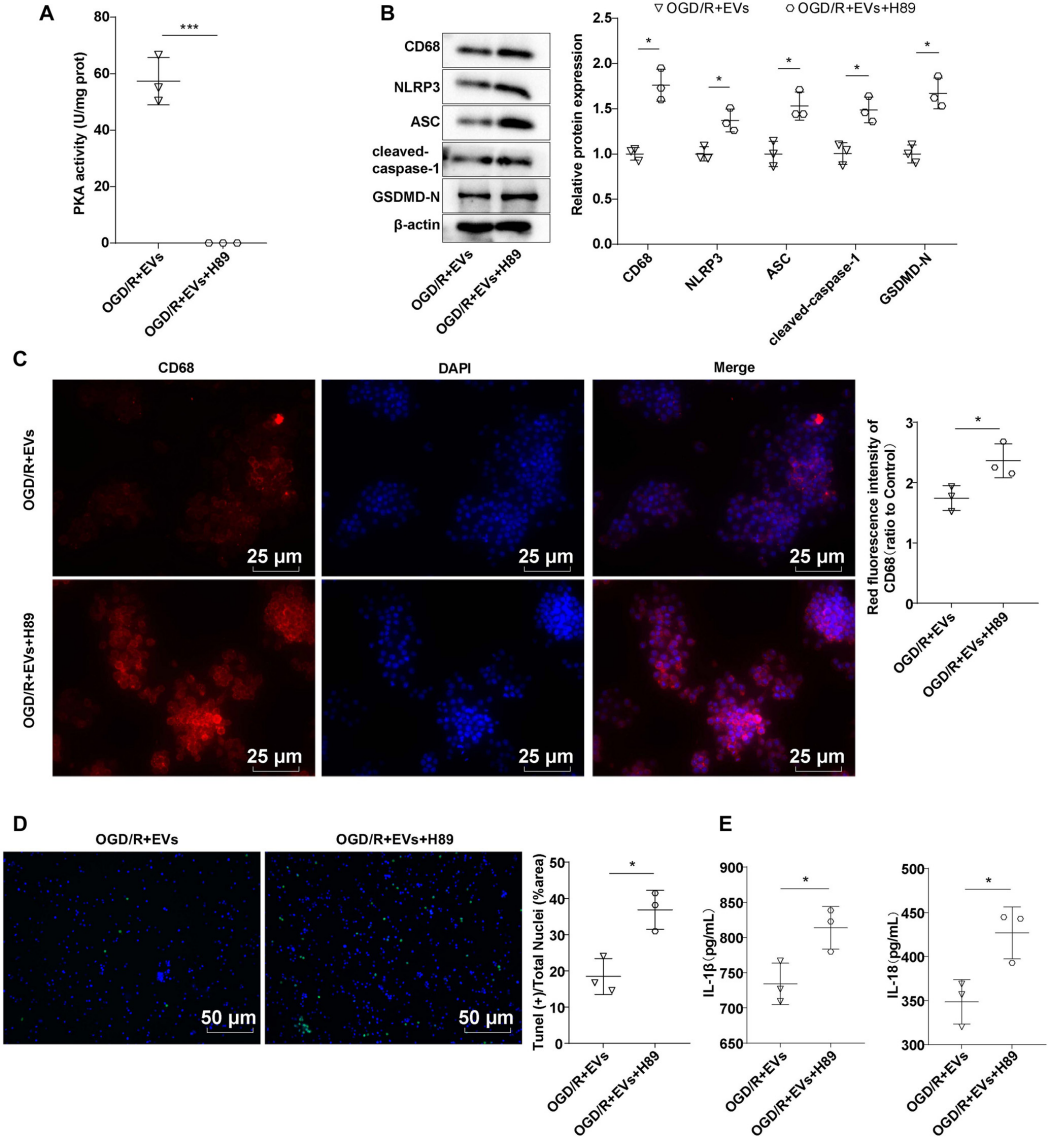
**Inhibition of PKA kinase activity partially reversed the ameliorative effect of hUC-MSC-EVs on OGD/R-induced pyroptosis of rat microglia.** (A) PKA kinase activity was detected by the kits; (B) The levels of CD68, NLRP3, ASC, cleaved caspase-1, and GSDMD-N were detected using Western blotting; (C) The level of CD68 was detected using immunofluorescence. CD68: Red; DAPI: Blue; (D) The number of dead cells in the hippocampus was detected by TUNEL staining. TUNEL positive: Green; DAPI: Blue; (E) The levels of inflammatory cytokines IL-18 and IL-1β were detected using ELISA. Cell experiments were repeated three times independently. Data were expressed as mean ± SD. *P < 0.05, ***P < 0.001. hUC-MSC-EV: Extracellular vesicles from human umbilical cord mesenchymal stem cells; DAPI: 4’,6-diamidino-2-phenylindole; ELISA: Enzyme-linked immunosorbent assay; OGD/R: Oxygen–glucose deprivation/reoxygenation; PKA: Protein kinase A; NLRP3: NOD-like receptor family pyrin domain-containing 3; IL-18: Interleukin-18; IL-1β: Interleukin-1β; TUNEL: Terminal deoxynucleotidyl transferase dUTP Nick end labeling; SD: Standard deviation; GSDMD-N: N-terminal cleaved gasdermin-D.

Cells were simultaneously treated with the PKA-selective inhibitor H89 and hUC-MSC-EVs. The OGD/R + EVs + H89 group showed no detectable PKA kinase activity, confirming that H89 effectively inhibited PKA kinase activity (Figure 6A, *P* < 0.001). Western blotting analysis revealed higher CD68 levels in the OGD/R + EVs + H89 group compared to the OGD/R + EVs group (Figure 6B, *P* < 0.05). Similarly, immunofluorescence showed increased CD68 fluorescence intensity in the OGD/R + EVs + H89 group compared to the OGD/R + EVs group (Figure 6C, *P* < 0.05). TUNEL staining indicated that treatment with H89 plus hUC-MSC-EVs resulted in a greater number of cell deaths (green fluorescence) compared to treatment with hUC-MSC-EVs alone (Figure 6D, *P* < 0.05). Moreover, the downregulation of PKA activity partially negated the inhibitory effects of hUC-MSC-EVs on NLRP3 inflammasome-related proteins and pyroptosis-related proteins (Figure 6B, *P* < 0.05). ELISA showed that simultaneous treatment with H89 and hUC-MSC-EVs led to higher IL-18/IL-1β levels compared to hUC-MSC-EVs alone (Figure 6E, *P* < 0.05). These findings suggest that the PKA inhibitor H89 partially reversed the protective effects of hUC-MSC-EVs on OGD/R-induced pyroptosis in microglia, further underscoring the critical role of the PKA signaling pathway in the anti-inflammatory mechanisms of hUC-MSC-EVs.

## Discussion

HIBD remains a major cause of neonatal morbidity and mortality, particularly impacting infants who experience a lack of oxygen and blood flow to the brain during the perinatal period. Despite significant advances in neonatal care, therapeutic options for HIBD are limited, with hypothermia being the most widely used intervention [58]. However, hypothermia offers only partial efficacy, leaving many infants with long-term neurological deficits, including cerebral palsy, cognitive impairments, and epilepsy [59]. These challenges underscore the urgent need for novel therapeutic strategies that address the underlying mechanisms of brain injury and promote neuroprotection. In this study, we investigated the therapeutic potential of hUC-MSC-EVs as a neuroprotective treatment for HIBD. Our findings demonstrated that hUC-MSC-EVs significantly reduced NLRP3 inflammasome activation, decreased microglial pyroptosis, and enhanced neuronal survival in a neonatal rat model of HIBD (Figure 7). These results provide important insights into the potential application of hUC-MSC-EVs as a therapeutic intervention. HIBD triggers a cascade of pathological processes, including excitotoxicity, oxidative stress, and, notably, neuroinflammation. Microglial activation is one of the earliest and most significant events in the inflammatory response to brain injury [60]. When activated, microglia adopt a pro-inflammatory phenotype and release cytokines, chemokines, and other mediators that exacerbate neuronal damage. Among these, the NLRP3 inflammasome plays a central role by promoting the release of IL-1β and IL-18, two cytokines that intensify brain injury [61]. Our study confirmed the pivotal role of the NLRP3 inflammasome in HIBD pathogenesis. We observed significantly elevated levels of NLRP3, ASC, and caspase-1 in the brains of untreated HIBD rats [62, 63]. These findings align with prior research indicating that NLRP3 activation induces pyroptosis—an inflammatory form of programmed cell death characterized by cell swelling, membrane rupture, and the release of pro-inflammatory intracellular contents. Pyroptosis amplifies neuroinflammation, creating a vicious cycle of cell death and inflammation that further aggravates brain injury. As such, targeting this pathway represents a critical therapeutic objective in mitigating the progression of HIBD.

Despite the established role of the NLRP3 inflammasome in HIBD, effective therapeutic strategies to inhibit its activation remain limited. Pharmacological inhibitors have shown promise in preclinical models [64], but their systemic effects and potential side effects hinder clinical use, particularly in fragile neonatal populations. hUC-MSC-EVs present a novel, cell-free therapeutic option, offering a natural approach to modulating inflammation without the risks of systemic drug administration. These nano-sized vesicles, naturally enriched with bioactive molecules, such as proteins, lipids, and nucleic acids, can influence cellular behavior [65]. Unlike cell-based therapies, which carry risks, such as immune rejection or uncontrolled cell proliferation, hUC-MSC-EVs provide a safer, more targeted means of delivering therapeutic cargo. In this study, we demonstrated that hUC-MSC-EVs significantly reduced neuronal apoptosis in the hippocampal CA1 region—one of the most vulnerable areas to hypoxic-ischemic injury [66]. This neuroprotective effect was accompanied by decreased microglial pyroptosis, indicating that hUC-MSC-EVs act through multiple complementary mechanisms. Future research should investigate the broader impact of hUC-MSC-EVs on interconnected pathways to better understand their therapeutic potential for mitigating neonatal HIBD.A key finding of our study was the ability of hUC-MSC-EVs to inhibit NLRP3 inflammasome activation. Specifically, we observed a marked downregulation of NLRP3, ASC, and caspase-1 expression in HIBD rat brains treated with hUC-MSC-EVs compared to untreated controls [67]. This suggests that hUC-MSC-EVs directly modulate the inflammasome pathway, thereby dampening the inflammatory cascade that leads to neuronal death. The reduction in IL-1β and IL-18 levels further supports their anti-inflammatory effects, as these cytokines are pivotal downstream mediators of NLRP3 activation [68]. Additionally, *in vitro* experiments using OGD/R to mimic ischemic conditions revealed that hUC-MSC-EVs reduced pyroptotic cell death in microglial cultures. This was evidenced by lower levels of cleaved gasdermin-D, a key protein in pyroptosis pore formation [69], and reduced caspase-1 activity. Notably, hUC-MSC-EVs also promoted microglial polarization toward an anti-inflammatory M2 phenotype, indicating their role in fostering a reparative microenvironment beyond merely inhibiting cell death. While the precise molecular mechanisms by which hUC-MSC-EVs inhibit the NLRP3 inflammasome remain unclear, our findings provide valuable insights. Previous studies suggest that PKA activation may regulate the ubiquitination and degradation of NLRP3, preventing its activation [70]. Although our study did not directly investigate PKA involvement, the observed reduction in NLRP3 levels following hUC-MSC-EV treatment raises the possibility that EVs enhance NLRP3 degradation through this or a related pathway. This aligns with earlier research demonstrating that MSC-derived EVs can modulate intracellular signaling pathways to promote repair and reduce inflammation [71]. For example, PKA activation has been associated with K63-linked ubiquitination of NLRP3, leading to its degradation and preventing activation. Additionally, PKA activation may indirectly influence AMP-activated protein kinase (AMPK) signaling, further regulating NLRP3 degradation and inhibiting pyroptosis [72]. Future studies should aim to identify the specific cargo within hUC-MSC-EVs responsible for these effects. Prior research has highlighted miRNAs, such as miR-21 and miR-146a, as key regulators of inflammasome activity, suggesting that hUC-MSC-EVs may deliver similar miRNAs to target cells in the brain [73, 74]. Proteomic analyses of EVs may also uncover proteins involved in inflammasome inhibition, such as those that enhance NLRP3 ubiquitination or block its assembly [75]. Advanced methods like RNA sequencing and proteomics will be critical to elucidate the molecular components mediating the therapeutic effects of hUC-MSC-EVs.

While our findings strongly support the neuroprotective effects of hUC-MSC-EVs in HIBD, several limitations must be addressed. First, the long-term efficacy of hUC-MSC-EV treatment remains uncertain. Although we observed significant reductions in inflammation and cell death during the acute phase of injury, it is unclear whether these effects translate into sustained functional improvements. Future studies should incorporate behavioral assessments to evaluate cognitive and motor outcomes in treated animals over extended periods. Moreover, the detection of cleaved caspase-1 in our study is not exclusively linked to microglia, as it may also be present in apoptotic neurons or other glial cell types [76]. This complicates the interpretation of pyroptosis-related findings, as neurons and other cells may contribute to the observed increases in cleaved caspase-1. Additional research is needed to identify the specific cellular sources of cleaved caspase-1 and to explore distinct pyroptotic mechanisms in neurons and microglia, thereby clarifying the pathways involved in HIBD. Another limitation is the timing of hUC-MSC-EV administration. In this study, we administered hUC-MSC-EVs only within a few hours of HIBD induction. Extending the treatment period may enhance therapeutic efficacy. Additionally, while we focused on the hippocampal region due to its high vulnerability to ischemic injury, future studies should examine the broader effects of hUC-MSC-EVs on other brain regions affected by HIBD. The specific molecular cargo within hUC-MSC-EVs responsible for their therapeutic effects was also not identified in this study. Future research should aim to isolate and characterize the miRNAs, proteins, and other bioactive molecules within hUC-MSC-EVs that contribute to their anti-inflammatory and neuroprotective functions. Identifying these components will be critical for optimizing EV-based therapies and ensuring reproducibility across different experimental models. Finally, while our results suggest that PKA activation plays a key role, its potential interaction with AMPK signaling, which regulates NLRP3 ubiquitination [72], was not explored. Investigating this pathway could provide greater insight into the molecular mechanisms underlying hUC-MSC-EV effects. Similarly, we did not evaluate oxidative stress or other inflammatory pathways [77, 78], which may also contribute to the therapeutic effects of hUC-MSC-EVs in HIBD. Despite these limitations, our findings highlight the potential of hUC-MSC-EVs as a therapeutic tool for addressing neuroinflammation and promoting neural repair. These findings have important clinical implications for the treatment of HIBD and other neuroinflammatory conditions. hUC-MSC-EVs represent a promising, non-invasive, cell-free therapeutic approach capable of modulating key inflammatory pathways. Compared to stem cell transplantation, EVs carry a lower risk of immunogenicity and tumor formation, making them a safer alternative for clinical use, particularly in neonates [79]. Beyond HIBD, the anti-inflammatory and neuroprotective properties of hUC-MSC-EVs could be explored in other neurological conditions characterized by excessive inflammation, such as traumatic brain injury, multiple sclerosis, and Alzheimer’s disease [80–82]. The ability of EVs to cross the blood–brain barrier and deliver therapeutic cargo directly to damaged brain regions positions them as a versatile tool for treating CNS disorders [83]. Future research should prioritize translating these preclinical findings into clinical trials to assess the safety and efficacy of hUC-MSC-EVs in human patients.

## Conclusion

In conclusion, this study presents compelling evidence for the neuroprotective effects of hUC-MSC-EVs in a neonatal rat model of hypoxic-ischemic brain injury. By targeting the NLRP3 inflammasome and reducing microglial pyroptosis, hUC-MSC-EVs demonstrate a promising therapeutic potential to mitigate the long-term consequences of HIBD. While additional research is needed to fully uncover the molecular mechanisms behind these effects and evaluate their long-term efficacy, the findings highlight the promise of hUC-MSC-EVs as a non-invasive, cell-free therapy for neuroinflammatory conditions. This study lays a solid foundation for future exploration into the clinical applications of hUC-MSC-EVs in neonatal brain injury and other related disorders.

## Supplemental data

**Graphical abstract.**
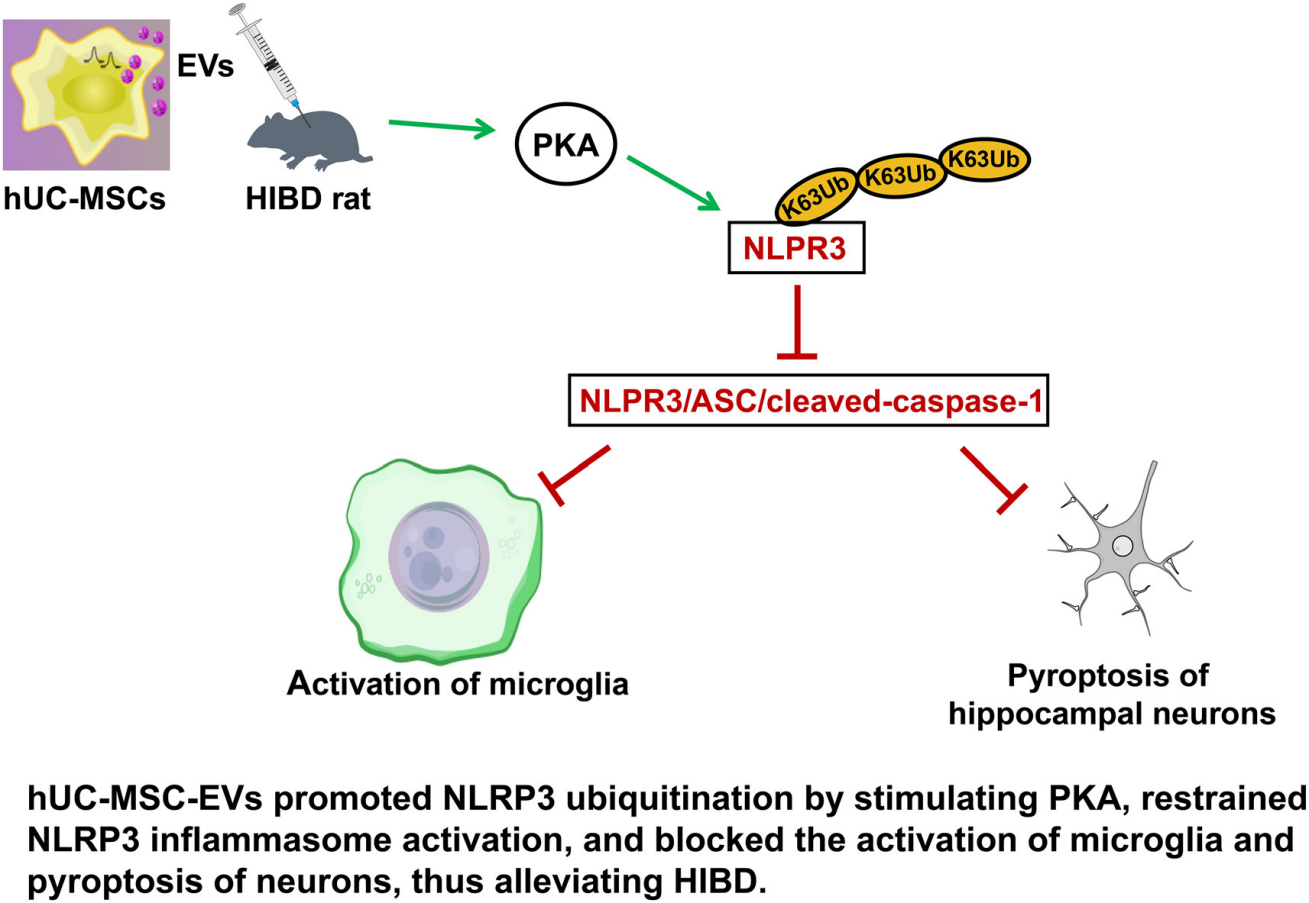
Graphical abstract of hUC-MSC-EVs reducing inflammation, promoting neuron survival, and modulating NLRP3 ubiquitination via PKA activation.

## Data Availability

All data generated or analyzed during this study are included in this article. Further enquiries can be directed to the corresponding author.
